# Neural Bases of Cognitive Impairments in Post-Traumatic Stress Disorders: A Mini-Review of Functional Magnetic Resonance Imaging Findings

**DOI:** 10.3389/fpsyt.2020.00176

**Published:** 2020-03-17

**Authors:** Gabriele Dossi, Giuseppe Delvecchio, Cecilia Prunas, Jair C. Soares, Paolo Brambilla

**Affiliations:** ^1^Department of Neurosciences and Mental Health, Fondazione IRCCS Ca’ Granda, Ospedale Maggiore Policlinico, Milan, Italy; ^2^Department of Pathophysiology and Transplantation, University of Milan, Milan, Italy; ^3^Department of Psychiatry and Behavioural Sciences, UT Houston Medical School, Houston, TX, United States

**Keywords:** post-traumatic stress disorder, cognition, fMRI, selective attention, response inhibition, memory

## Abstract

**Introduction:**

Post-Traumatic Stress Disorder (PTSD) is often associated with impairments in emotional and cognitive domains. Contrarily to the emotional sphere, neural basis underpinnings to cognitive impairments are still not well known.

**Methods:**

We performed a bibliographic search on PUBMED of all the studies investigating the cognitive impairments in PTSD individuals. We considered only studies that applied cognitive tasks using a functional Magnetic Resonance Imaging technique. The inclusion criteria were met by nine studies.

**Results:**

Overall, PTSD individuals reported significant impairments in the dorsolateral prefrontal cortex, anterior cingulate cortex, inferior frontal gyrus, insula, inferior temporal cortex, supplement motor area, and Default Mode Network (DMN). Moreover, abnormal activity was reported in subcortical structures (e.g. hippocampus, amygdala, thalamus) and in the cerebellum.

**Limitations:**

Cognitive functioning was assessed using different cognitive tasks. Potential confounding factors such as age, sex, symptoms intensity, and comorbidities might have influenced the results.

**Conclusion:**

So far, the evidence reported that PTSD is characterized by cognitive impairments in several domains, such as attention, memory and autonomic arousal, which may be due to selective dysfunctions in brain regions that are part of cortical networks, the limbic system and DMN. However, further studies are needed in order to better assess the role of cognitive impairments in PTSD and to develop more targeted therapeutic approaches.

## Introduction

Post-Traumatic Stress Disorder (PTSD) is defined by the Diagnostic and Statistical Manual of Mental Disorder Fifth Edition (DSM-5) as a psychiatric disorder that can occur in people after experiencing a traumatic event, such as cataclysms, severe accidents, terroristic attacks or brutal personal assaults (1). Moreover, PTSD can occur in all people regardless of age, ethnicity, nationality, and culture, with women twice as likely as men to develop PTSD (1).

Moreover, PTSD is characterized by several symptoms, including intrusive thoughts, avoiding behaviors, negative thoughts and hyperarousal symptoms, such as irritability, sleep disorders and hypervigilance, which may, in turn, cause impairments in several cognitive domains, such as memory, attention and autonomic arousal (2). Therefore, recently, several studies on PTSD have explored the different cognitive domains using neuropsychological tests and neuroimaging techniques, including structural and functional magnetic resonance imaging (MRI) (2, 3). To the best of our knowledge, the majority of these studies focused on cognition in relation to emotion, using tasks with emotional valence stimuli (e.g. recalling traumatic event, emotion recognition tasks). Specifically, the evidence reported by neuropsychological studies showed that PTSD patients had enhanced memory performances in recalling events and items with negative emotional valence, enhanced responsivity to fear conditioning and increased attentional bias in processing threat stimuli (2). Additionally, it has also been reported that PTSD subjects were characterized by slowed goal-direction activity, impairments in recalling and learning neutral information, inability in extinction learning, as well as difficulties in remembering specific neutral and autobiographical events (2). Interestingly, in recent years, neuroimaging investigations have provided important insight on the neurobiological underpinnings of these cognitive deficits in PTSD patients. Specifically, structural and resting-state MRI studies on PTSD reported gray matter and connectivity alterations in cortical areas, such as anterior cingulate cortex (ACC), prefrontal cortex (PFC) and insula, as well as in subcortical structures, such as hippocampus and amygdala (4), all regions known to be part of a network which regulates contextual processing (5). Finally, disproportional hypervigilance and tendency to interpret neutral or safe situations as dangerous have also been considered hallmarks of PTSD (5), further supporting the presence of a dysregulation in the contextual processing network in PTSD patients, which in turn may explain impairments in modulating fear inhibition, emotion and attention regulation, as well as autonomic responses (3, 6, 7).

However, although the relation between cognition and emotion in PTSD has been well explored, to our knowledge, the study of cognitive mechanisms and their neural correlates detached from the emotional sphere is more limited. Notably, several findings showed that cognitive and emotional networks are differently affected in PTSD subjects, suggesting that cognitive and emotional domains, although strictly intertwined, need to be investigated separately (8, 9). For these reasons, in the last years, there has been an increased interest in the investigation of neuropsychological and cognitive features characterizing PTSD (2), especially for identifying the role of stress and anxiety in influencing normal cognitive processing. The evidence emerging from these studies is paramount to understanding the neural basis of PTSD, its development and its treatment, since they are critical for the social, occupational and emotional functioning (10). In this context, this review aims at summarizing all functional MRI studies (fMRI) that investigated the neural bases of cognitive impairments in PTSD using cognitive tasks.

## Materials and Methods

We carried out a bibliographic search in PubMed and Scopus using “*PTSD AND fMRI AND cognition*” and “*PTSD AND fMRI and EMOTION*” and “*PTSD AND fMRI AND cognition AND deficits*”. We also used “*EMOTION*” as a keyword because in many studies cognitive domains were explored together with emotional aspects. However, for this review, we only (treatment as usual) selected the fMRI studies in which cognition was investigated outside the context of an emotional task. No time restrictions were used and we selected studies until February 2019. We excluded studies that a) employed neuroimaging techniques other than fMRI, including resting-state fMRI and real-time fMRI, b) used tasks with emotional valenced stimuli only (e.g. faces, pictures or sounds with affective contents), c) were not in humans, d) explored PTSD in relation to personality disorders, e) investigated at-risk subjects for PTSD disorder. The inclusion criteria were met by nine studies whose methods and results are summarized in **Table 1**. Specifically, for the cognitive domain explored, seven studies focused on attention and response inhibition (11, 12, 14, 16–19), one study explored memory functions (13), and one study investigated autonomic arousal (15).

**Table 1:** Demographic and clinical characteristics of the fMRI studies included in the review (alphabetical order).

Study	Participants (Male/Female)	Age Mean, years (SD)	Design	MRI sequence	Clinical scale	edications	Comorbidity	Main results
**Aupperle et al**. (11)	10 PTSD (0/10)	PTSD: 34.60 (9.40)	Stop Signal Block	fMRI (3.0T)	PCL CAPS D-KEFS	No medication	Not specified	**PTSD > HC** R Middle Frontal (dorsolateral PFC) R Superior Temporal/Insula
	12 HC (0/12)	HC: 35.25 (6.44)						
								**PTSD < HC** R Inferior Parietal
								L Superior medial PFC
								R Rostral ACC
								R Putamen
								L Caudate and Putamen
								R Middle Temporal
								R Parahippocampal gyrus
								L Postcentral gyrus
								R/L Precuneus
								L Paracentral Cortex
								L Middle Occipital
								R Lingual gyrus
								L Cerebellar Tonsil
								R Superior Cerebellum
**Bluhm et al**. (12)	20 PTSD (7/13)	PTSD: 39.35 (9.43)	Self Referential Processing	fMRI (4.0T)	CAPS SCID CTQ	No medication	7/20 MDD 5/20 Current MDE 3/20 Dysthimia 1/20 Panic Disorder 1/20 Agoraphobia 7/20 GAD 5/20 Social Anxiety Disorder 1/20 Specific Phobia 1/20 OCD	Self-relevance vs General facts **HC > PTSD** dorsal medial PFC ventral medial PFC PCC/Precuneus **PTSD > HC** L dorsal medial PFC Precuneus
	15 HC (2/13)	HC: 37.33 (11.52)	Block					
**Carrion et al**. (13)	16 PTSS (6/10)	PTSS: 13.9 (2.0)	Verbal Declarative Memory	fMRI (3.0T)	CAPS-CA CBCL K-SADS WAIS	No medication	3/16 Depressive Disorder not specified	Retrieval task **PTSS < HC** Bilateral Hippocampus
	11 HC (7/4)	HC: 13.9 (1.9)	Block				3/16 MDD 1/16 Panic Disorder 1/16 Enuresis 1/16 ADHD+C.D.+Social Phobia	
**Clausen et al**. (14)	39 PTSD (39/0)	PTSD: Not specified	Multisource Interference	fMRI (3.0T)	PCL-Military Version	Not specified	Not specified	Congruent Blocks Rostral ACC Rostral medialPFC Ventral ACC Incongruent Blocks dorsal medial PFC dorsal ACC
			Block		CAPS			
					MINI			
					D-KEFS			
					Tower Test			
					SDMT			
					AVLT			
					TMT			
					NAB Digits			
					WTAR			
**Felmingham et al**. (15)	11 PTSD (9/2)	PTSD: Not specified	Auditory Oddball	fMRI (1.5T)	CAPS SCID DASS	4/11 Antidepressants	7/11 Depression 1/11 Panic Disorder	**PTSD > HC** R rostral ACC Bilateral Hippocampus R Supramarginal gyrus R Superior Frontal Cortex R Fusiform gyrus L Thalamus R middle Frontal gyrus R Parahyppocampal gyrus R supplementary motor area L middle Cingulate **HC > PTSD** No significant clusters
	11 HC (9/2)	HC: Not specified	Event-Related					

**Jovanovic et al**. (16)	20 PTSD+ (0/20)	PTSD+: 36.6 (3.3)	Go/No-Go Event-Related	fMRI (3T)	Modified PSS CTQ TEI STAI	No medication	Not specified	**PTSD- > PTSD+** Ventromedial PFC/rostral ACC **PTSD- < PTSD+** No significant clusters
	21 PTSD- (0/21)	PTSD-: 39.8 (2.8)						
**Scheibel et al**. (17)	7 PTSS + (Not specified) 8 PTSS - (Not specified)	PTSS +: 31 (5) PTSS -: 30.88 (6.36)	Arrow Task Event-Related	fMRI (3T)	P.C.L. - Civilian BSI NSI Barona IQ	PTSS+ Antidepressants: 4/7Mood Stabilizers 1/7 Pain Medication 3/7 Sleep aids 1/7 PTSS- Antidepressants: 3/8 Pain Medication 4/8 Sleep Aids 1/8	Not specified	**PTSS- > PTSS+** Bilateral Cerebellum Bilateral Basal ganglia Bilateral Cingulate gyrus Bilateral Cuneus **PTSS+ > PTSS-** No significant clusters
**Thomaes et al**. (18)	29 PTSD (0/33) B.L.	PTSD: 33.5 (11.6)	Classic Stroop Task Block **Treatment** TAU: Supportive care and /or pharmacotherapy EXP: Psycho-educational + Cognitive Behavioural group therapy + TAU	fMRI (1.5T)	STI CAPS BDI DES BPDSI SCID-D	19/29: SSRI 15/29: Anxiolytics	21/29: Anxiety Disorders	**BASELINE** **PTSD > HC** Bilateral Superior Frontal gyri R Parahippocampal gyrus R Fusiform gyrus L anterior Insula R dorsal ACC PPC Bilateral Premotor cortex/SMA **PTSD < HC** No significant clusters **AFTER TREATMENT** **EXP post > pre** No significant clusters **EXP post < pre** Bilateral dorsal ACC
	22 HC (0/30) B.L.	HC: 35.2 (9.9)					18/29: Depressive disorders	
	16 PTSD (0/16) A.T.	PTSD: Not specified						

								R Superior frontal gyrus
								L Anterior Insula
								Premotor Cortex/SMA
								**TAU post > pre:** No significant clusters
								**TAU post < pre** No significant clusters
**van Rooij et al**. (19)	28 PTSD (28/0)	PTSD: 36.6 (10.6)	Stop Signal Anticipation	fMRI (3.0T)	CAPS SCID DSS	Anxiolitycs 1/28	15/28: Mood disorders 7/28: Anxiety disorders 2/28: Somatoform/Pain disorders	PTSD < CC, HC (Proactive Inhibition): rostral IFG
	26 CC (26/0)	CC: 37.2 (10.1)	Block					
	25 HC (25/0)	HC: 34.8 (9.5)						

PTSD, Post Traumatic Stress Disorder; PTSD+, Post Traumatic Stress Disorder with higher symptoms; PTSD-, Post Traumatic Stress Disorder with lower symptoms; PTSS, Post Traumatic Stress Symptoms; HC, Healthy Control; CC, Combat Control; B.L., Baseline; A.T, After Treatment; PCL, PTSD Checklist; CAPS, Clinical Administered PTSD Scale; CAPS CA, CAPS for Children/Adolescent; D-KEFS, Delian Kaplan Executive Function System; SCID, Structural Clinical Interview; CTQ, Childhood Trauma Questionnaire; CBCL, Child Behavior Checklist; K-SAD, Kidde Schedule for Affective Disorder; WAIS, Weschler Adult Intelligence Scale; MINI, Mini International Neuropsychiatric Interview; SDMT, Symbol Digit Modalities Test; AVLT, Auditory Verbal Learning Test; TMT, Trail Making Test; NAB, Neuropsychological Assessment Battery; WTAR, Weschler Test of Adult Reading; DASS, Depression, Anxiety and Stress Scale; TEI, Traumatic Events Inventory; STAI, State Trait Anxiety Inventory; BSI, Brief Symptoms Inventory; NSI, Neurobehavioral Symptom Inventory; STI, Structured Trauma Interview; BDI, Beck's Depression Inventory; DES, Dissociative Experience Scale; BPDSI, Borderline Personality Disorder Severity Index; DSS, Dissociative Symptom Scale; R, Right; L, Left; PFC, PreFrontal Cortex; ACC, Anterior Cingulate Cortex; PCC, Post Cingulate Cortex; SMA, Supplement Motor Area; IFG, Inferior Frontal Gyrus; TAU, Treatment As Usual; EXP, Experimental Treatment; SD, Standard Deviation.

## Results

Most of the results refer to two cognitive domains, selective attention and response inhibition, which were explored in subjects affected by PTSD through different cognitive tasks.

Specifically, the first fMRI study was performed by Thomaes et al. (18) who carried out a longitudinal study to investigate the impact of 6 months psycho-educational and cognitive behavioral stabilizing treatment in addition to the classic PTSD therapy (experimental treatment), or of classic therapy only (treatment as usual) on selective attention in PTSD patients compared to healthy controls (HC) using a Classic Stroop test. Interestingly, at baseline, the authors found that both PTSD patients and HC showed greater activations in the inferior frontal gyrus (IFG), extending to the Broca’s area, dorsal ACC, supplement motor area (SMA), posterior parietal cortex, secondary visual cortex bilaterally, inferior temporal cortex and insula. Additionally, PTSD individuals also reported greater activations in the left inferior insula and dorsal ACC compared to HC. Although no differences were observed between medicated and non-medicated patients, the authors found that patients with co-morbid major depressive disorder (MDD) displayed reduced activation in dorsal ACC compared to non-MDD patients. Moreover, at follow-up, the whole group of PTSD patients, regardless of treatment, improved the performance in the Stroop test in terms of accuracy, with subjects in the experimental therapy also showing shorter reaction times. In addition, during the task, while both groups showed a reduced activation in the premotor cortex/SMA and left inferior frontal cortex at the end of the study period, patients that followed the experimental treatment revealed decreased responses in bilateral dorsal ACC, left anterior insula and superior PFC compared to the classic therapy group.

Furthermore, in a Go/No-Go task, Jovanovic et al. (16) investigated attention and response inhibition in a sample of 41 women. Among them, 20 showed higher current PTSD symptoms (PTSD+) as opposed to the healthy remaining 21 (PTSD-). Interestingly, the behavioral responses during the Go and No-Go trials were very accurate in both groups, with no differences in the error rate. However, during the fMRI task the authors found that the PTSD+ group showed reduced activation in the ventromedial PFC compared to the PTSD- group. Additionally, attention and inhibition impairments were also explored by van Rooij et al. (19) using the stop-signal anticipation task (SSAT), a modified version of the Stop Signal Task (20), which was employed for exploring reactive and proactive inhibition in a sample of 28 PTSD male veterans, 26 male veterans without current psychiatric illnesses (combat control, CC), and 25 HC. Specifically, the authors defined reactive inhibition as the outright stopping of a response managed by motor areas, whereas proactive inhibition was defined as the anticipation of stopping, relying on the processing of contextual clues. With respect to reactive inhibition, speed inhibition was faster in HC than PTSD and combat control (CC) groups. Moreover, PTSD patients showed less reduction in activation (inhibition) in the left pre/post central gyrus compared to CC and HC groups during the SSAT. However, all groups activated neural networks usually involved in response inhibition, such as the right IFG, insula, right supramarginal gyrus, right SMA and left superior frontal gyrus, as well as deactivating the default mode network (DMN). Regarding proactive inhibition, although response time data showed that the PTSD group had reduced inhibition compared with CC and HC groups, no brain activation differences were observed between the groups during the SSAT.

Interestingly, similar results were found by Scheibel et al. (17) who investigated response inhibition and attention in 15 veterans using a modified version of the Arrows Task. During this task, the participants viewed blue or red arrows for 265 milliseconds (ms), and each was then followed by a blank screen for 200 ms and a cross hair fixation point for another approximately 2235 ms. Subjects were required to respond with a button press on the same side as the one in which the arrows were pointing when the arrows were blue, and to the opposite side when the arrows were red (17, 21). Based on the PTSD Checklist (PCL), the sample was split in 7 subjects with relatively high PTSD symptoms (HIGH PTSS; PCL≥39) and in 8 subjects with lower or no symptoms (LOW PTSS; PCL ≤ 31). The full sample analysis revealed deactivation of the posterior dorsal anterior cingulate gyrus, the right lateral frontal lobe, bilateral parietal structures and the precentral gyrus. In addition, the LOW PTSS group showed hyper-activations within both temporal lobes, the left cingulate and precentral gyri, the right thalamus and parts of the basal ganglia. Conversely, the HIGH PTSS group showed no significant activations but only extensive deactivation in cortical areas (e.g. left cingulate gyrus), subcortical structures (e.g., left amygdala, bilateral caudate), and bilateral cerebellum. Moreover, the comparison between LOW PTSS and HIGH PTSS patients highlighted a greater activation of bilateral large areas of the occipital, parietal and temporal lobes (e.g. angular gyrus, cuneus, fusiform gyrus), cerebellum, basal ganglia (e.g. left putamen), anterior and posterior cingulate gyri and the right lateral PFC within LOW PTSS individuals. Notably, there were no areas in which HIGH PTSS individuals had greater activation than LOW PTSS individuals.

Furthermore, Aupperle et al. (11) explored the neural correlates of response inhibition in a sample of 10 female PTSD patients and 12 HC with the stop signal task. The comparison between the two groups revealed greater activation in the right dorsolateral PFC, right superior temporal gyrus and the right anterior insula in the PTSD group compared to HC. Moreover, the PTSD group showed less differential activation in several DMN regions (e.g. precuneus, medial PFC). On the other hand, the HC group had more activation than the PTSD group in the left IFG, the right rostral ACC, and the lateral middle frontal gyrus than PTSD subjects. Further, HC revealed less differential activation within the anterior insula and the posterior cingulate cortex (PCC) than PTSD individuals.

Additionally, the fMRI study carried out by Clausen et al. (14) used a Multisource Interference task (MSIT) in a cohort of 39 male veterans with different levels of PTSD. Comparably to the above-mentioned findings, the authors showed that ventral ACC, rostral ACC and rostral medial PFC resulted in more activation during congruent trials, whereas incongruent trials elicited greater dorsal ACC and dorsomedial PFC activation. Moreover, the authors showed that worse PTSD symptoms were related to less rostral ACC activation. A reduced functional connectivity was found between both rostral ACC and medial PFC and lateral PFC regions. Finally, a particular form of selective attention was explored by Bluhm et al. (12) who investigated the neural bases of self-referential processing (SRP) in a sample of 20 PTSD subjects and 15 HC using an SRP task (12). The results from the within-group analyses showed that PTSD individuals had greater response in left dorsomedial PFC and in the precuneus, whereas HC had reduced activation in ventromedial PFC, dorsomedial PFC, PCC, and precuneus in the processing of self-knowledge vs. general facts contrast. Moreover, the between-group comparisons revealed that PTSD patients had reduced ventromedial PFC activation in response to self-knowledge vs. general facts compared to HC.

With regards to other cognitive domains, the last two fMRI studies included in this review explored memory and autonomic arousal in PTSD patients compared to HC.

Specifically, for the memory domain, Carrion et al. (13) investigated the role of hippocampal activity in 16 adolescents with Post-Traumatic Stress Symptoms (PTSS) and 11 adolescents HC. All subjects performed a Verbal Declarative Memory Task with encoding and retrieval trials while undergoing fMRI scanning. The comparison between PTSS and HC during encoding trials showed no difference in hippocampal activation, whereas HC groups exhibited a greater activation of the right hippocampus during retrieval trials compared to PTSS. Furthermore, the authors also found a significant correlation between PTSD symptoms, specifically avoidance and numbing, and the reduction of left hippocampal activity in PTSS subjects.

Finally, Felmingham et al. (15) investigated the neural correlates associated with autonomic arousal in 11 PTSD subjects and 11 HC in response to salient stimuli in an Auditory Oddball task (AOT). The authors explored the Orienting Responses (ORs) that are critical markers of the registration of new or significant stimuli and mobilization of attention and motor responses (22). The authors used the skin conductance response (SCR), which is a powerful index of the OR since it is elicited by unexpected and potential threatening stimuli. In this regard, “oddball” stimuli (with SCR) can be considered as sudden sensory changes, as opposed to the frequent standard stimuli (without SCR). In light of this, the authors explored the response to targets both with SCR and without SCR. In relation to averaged analysis (target-background), PTSD individuals showed more enhanced activation than HC in right rostral ACC, bilateral hippocampus, right supramarginal gyrus, right superior frontal cortex, right fusiform gyrus, left thalamus, right middle frontal gyrus, right parahippocampal gyrus, right SMA, and left middle cingulate. These effects were confirmed in a subsequent analysis without individuals with depression or in treatment with antidepressants, which also revealed a major activation in right dorsal lateral frontal cortex in HC but no activity in hippocampus was found in the PTSD group. Also, the analysis between “with SCR-without SCR” targets showed that “with-SCR” targets engaged the right ventral ACC network, left supramarginal gyrus and the left inferior frontal cortex in HC. Specifically, greater activation was found in ventral ACC and in left inferior lateral frontal cortex in HC, compared to the PTSD group. In contrast, PTSD subjects showed a major engagement in dorsal ACC, right supramarginal gyrus and dorsolateral frontal regions bilaterally, displaying greater activity in bilateral dorsolateral PFC and in left supramarginal gyrus. Notably, these findings were replicated without considering depressed participants under treatment and the results showed that the PTSD group revealed greater activity in dorsal ACC than HC to “with-SCR” targets.

## Discussion

The majority of fMRI studies reviewed reported abnormal activations in executive functions, such as response inhibition and selective attention in PTSD patients. These functions are critical for developing a normal social and occupational life, as they have a key role in emotion regulation (10, 23). Indeed, response inhibition is the suppression of automatic responses to a situation that is no longer needed, and the appropriate adjustment of the behavioral response. Therefore, inhibition is fundamental in maintaining optimal cognitive functioning but also to suppress dysfunctional responses typical of PTSD, such as hypervigilance and intrusive thoughts (23). Similarly, impairments in selective attention contribute to developing and maintaining PTSD symptoms in several ways, such as strengthening the association between threat and stimuli during traumatic events and enhancing the detection of threatening stimuli (24).

Interestingly, from the abovementioned results emerged the hypothesis that prefrontal dysfunctions, especially in regions within the IFG, ACC, and medial/lateral PFC, might be considered key alterations characterizing PTSD patients while processing of response inhibition tasks. This is not surprising, especially because the dorsal regions of ACC and medial PFC seem to be implicated in cognitive and attention regulation (8, 25), whereas the IFG has often been associated with proactive response inhibition, a cognitive function that refers to the anticipation of stopping and relies on the processing of contextual cues (26, 27). Notably, the fMRI studies here reviewed also reported increased activation of amygdala and insula, two areas that are respectively involved in processing salient, external stimuli and in processing internal bodily states (28). Therefore, all these findings suggest that lower activation of ACC, medial and lateral PFC and IFG might reflect impairments in down-regulating amygdala and insula activation, which may ultimately lead to the cognitive deficits observed in PTSD patients (28, 29).

Moreover, several studies found alterations in the modulation of the DMN (11, 12, 14, 19), a network which remains very active when the brain is at rest and deactivates when cognitive performance is required (30, 31), which includes several brain regions, such as middle temporal lobe, PCC, medial PFC, inferior parietal lobule, and lateral temporal cortex (30–32). In recent years, interest in DMN has grown since both resting-state and functional MRI studies discovered its role in the consolidation of memory, processing of internal and external stimuli and, remarkably, in the interplay between emotional stimuli and their cognitive elaboration (31, 32). Therefore, the involvement of this network observed by the above-mentioned studies is not surprising since it suggests that PTSD individuals have difficulties in disengaging DMN during low demanding cognitive tasks, which may lead to impairments in modulating executive control during high demanding cognitive tasks. Notably, these results are also consistent with previous studies that highlighted DMN alterations in PTSD patients (33, 34). Specifically, Sripada et al. (33) found reduced functional connectivity between rostral ACC, dorsomedial PFC and hippocampus at rest. Moreover, the same areas resulted hypoactive during cognitive tasks both with and without trauma-relevant stimuli. Similar results were obtained by a study on working memory that reported abnormal reduced connectivity between the PCC, frontal, temporal and parietal regions (implicated in the switching from resting state to activation), and abnormal enhanced connectivity between medial PFC and bilateral parahyppocampal gyrus (34). Altogether, these findings suggest that PTSD patients are characterized by an inadequate DMN functional integration during resting-state and a general DMN alteration during goal directed tasks, which may in turn cause poor performance in cognitive tasks (33, 34).

Interestingly, two studies (14, 17) also reported correlations between symptoms severity and neuropsychological performance, as well as prefrontal, parietal and temporal activity, further supporting the hypothesis that the activation of regions involved in executive control (e.g. middle frontal gyrus, inferior parietal lobule, ACC) decreased in association with greater symptom severity (9, 35). Additionally, a further negative correlation between activation and symptoms was found in subcortical structures (e.g. thalamus) and in the cerebellum, which have well-known connections with the associative cortex (36). Therefore, these findings seem to suggest that the extension of the cognitive dysfunctions observed could be connected to the intensity of the symptoms. Therefore, although these results may still be limited, they aligned with the literature, reporting significant correlation between disease severity and cognitive impairments in psychiatric diseases, such as schizophrenia and bipolar disorder (37, 38).

Finally, two studies found impairments in the autonomic arousal (15) and in the memory domain (13) in PTSD patients.

Although deficits in autonomic arousal are known to be linked to PTSD (39), fMRI studies investigating the neural correlates of this symptom are yet to be fully explored. Nonetheless, the results obtained by Felmingham et al. (15), especially the increased activation in PTSD individuals of dorsal ACC, hippocampus and supramarginal gyrus, are not surprising since these areas are known to be involved in anxiety disorders (40). Finally, it has also been reported that PTSD patients had altered activity in the hippocampus while processing a memory task (13). This finding is not surprising, especially because a robust body of literature in the last decades has consistently reported the key role played by the hippocampus in declarative memory (encoding and retrieving information), semantic and episodic memory, as well as in emotional memory [for recent reviews, see (41, 42)]. Furthermore, this result is also supported by behavioral data, suggesting that subjects affected by PTSD have poor performance in memory tasks, including both neutral and emotional stimuli, as well as severe difficulties in retrieving neutral words and autobiographical episodes (42). Finally, several neuroimaging studies also reported smaller hippocampal volumes in individuals with PTSD and abnormal hippocampal activity both during encoding and retrieval processes (4, 43, 44), ultimately suggesting that the hippocampus is a fundamental structure for cognitive and emotional processes in PTSD.

### Limitations

This review should be considered in light of some limitations. First, brain activity and performance during fMRI scanning were assessed using different cognitive tasks, which may explain some heterogeneity in the results. Second, the sample considered in the reported studies was small and included patients with varying age, sex and symptoms’ intensity. Third, subjects involved in the studies had comorbidities with other psychiatric diseases, such as depression and anxiety disorders, which reduced the specificity of their findings. Fourth, the majority of the fMRI studies reviewed here employed PTSD patients under various pharmacological treatments. Importantly, the use of medication should be considered carefully since it is not a confounding factor per se, though its use can influence the BOLD signal both at a neural and vascular level in a manner that is not completely clear yet (45). However, it is important to mention that the majority of PTSD patients were undergoing treatment with various medications (e.g. antidepressant, anxiolytics, mood stabilizer), thus providing a more biologically realistic estimation of the brain in PTSD. Finally, it is also important to note that the number of fMRI studies exploring cognitive domains alone in PTSD are still sparse, ultimately limiting the knowledge of neural basis of cognitive impairments associated with this disorder.

### Conclusion

In conclusion, the present review suggests that PTSD seems to be characterized not only by emotional processing impairments, but also by cognitive disturbances. Specifically, PTSD patients showed abnormal activation mainly in dorsal medial PFC, IFG, ACC, hippocampus, as well as in regions that are part of the DMN, ultimately suggesting that this disorder is characterized by impairments in top-down cognitive processes involved in the modulation of external and internal stimuli response. However, future fMRI studies are warranted for an in-depth analysis of the putative biomarkers associated with cognitive symptoms often observed in this disorder. This is valid especially for resting-state MRI studies, since the investigation of functional connectivity deficits between regions and the identification of dysfunctional brain networks in PTSD patients will allow for more insights about this disease. All these strategic approaches may in turn permit the development of more targeted treatments and preventive approaches.

## Author Contributions

GDo and GDe wrote the manuscript. PB and CP participated in the revision and proof-reading process of the manuscript together with JS. All authors have approved the final manuscript.

## Funding

PB was partially supported by grants from the Italian Ministry of Health (RF-2016-02364582).

## Conflict of Interest

The authors declare that the research was conducted in the absence of any commercial or financial relationships that could be construed as a potential conflict of interest.
